# MiR-187-3p Enhances Propranolol Sensitivity of Hemangioma Stem Cells

**DOI:** 10.1247/csf.18041

**Published:** 2019-02-02

**Authors:** Chao Liu, Zeliang Zhao, Zhidong Ji, Yanyan Jiang, Jiawei Zheng

**Affiliations:** 1 Department of Oromaxillofacial Head and Neck Oncology, Shanghai Ninth People’s Hospital, College of Stomatology, Shanghai Jiao Tong University School of Medicine, Shanghai 200011, China; 2 Department of Oral and Maxillofacial Surgery, Shandong Provincial Hospital Affiliated to Shandong University, Jinan, Shandong 250012, China; 3 EasyFiber Technologies Inc, Jinan, Shandong 250012, China; 4 Key Laboratory for Liquid-Solid Structural Evolution and Processing of Materials, Ministry of Education, Shandong University, Jinan 250061, Shandong, China

**Keywords:** MiR-187-3p, infantile hemangioma, propranolol, resistance, NIPBL

## Abstract

Infantile hemangioma is the most common soft tissue tumors in childhood. In clinic, propranolol is widely used for infantile hemangioma therapy. However, some of the infantile hemangioma patients display resistance to propranolol treatment. Previous studies show that miR-187-3p is inhibited in hepatocellular carcinoma and lung cancer, while the role of miR-187-3p in infantile hemangioma remains unclear. In the present study, we explore the biological role of miR-187-3p in infantile hemangioma. The mRNA and protein levels of related genes were detected by real-time PCR and Western blotting. CCK8 assay was used to detect cell viability and IC50 values of propranolol. Cell apoptosis was detected by Caspase-3 Activity assay. Luciferase reporter assay and biotin RNA pull down assay were used to detect the interaction between miR-187-3p and the targeted gene. MiR-187-3p was down-regulated in infantile hemangioma tissues and promoted propranolol sensitivity of HemSCs. Mechanically, NIPBL was the direct target of miR-187-3p in HemSCs. NIPBL downregulation inhibited propranolol resistance of HemSCs. Re-introduction of NIPBL reversed miR-187-3p-meidated higher propranolol sensitivity of HemSCs. MiR-187-3p enhanced propranolol sensitivity of hemangioma stem cells via targeting NIPBL. MiR-187-3p may serve as a novel prognostic indicator and potential target for infantile hemangioma therapy.

## Introduction

Infantile hemangioma is one kind of benign vascular neoplasms and the most common soft tissue tumor in infancy. The incidence of infantile hemangioma in infancy is about 3% to 10% (Hoornweg *et al.*, 2005; Leaute-Labreze *et al.*, 2015). Infantile hemangioma develops quickly during the first year of childhood (proliferating phase), then grows slowly during 1–5 years (involuting phase), and eventually disappears by age 5–10 (involuted phase) (Boye *et al.*, 2001; Mulliken, 1993; Takahashi *et al.*, 1994). However, the cause of infantile hemangioma remains unclear. In clinic, propranolol is considered as safe and highly effective to treat infantile hemangioma, and is widely used as a first line therapy (Schupp *et al.*, 2011). However, a rare part (10/1130, 0.9%) of patients with infantile hemangioma would show resistance to propranolol treatment (Causse *et al.*, 2013). As hemangioma derived stem cells (HemSCs) was believed to be crucial for infantile hemangioma development (Bischoff, 2009; Greenberger *et al.*, 2010b), we aim to explore whether HemSCs are responsible for propranolol resistance.

MicroRNAs (miRNAs) are a kind of non-coding small RNAs, which play important roles in various biological processes via regulating the translation of its target genes (Liu, 2008; Zhao and Srivastava, 2007). In tumors, miRNAs were reported to have the potential of promising targets for the diagnosis and prognosis (Di Leva *et al.*, 2014). In non-small cell lung cancer (NSCLC), miR-187-3p expression levels were much lower in primary tumor tissues and NSCLC cell lines. MiR-187-3p overexpression promoted cell apoptosis, inhibited proliferation, migration and invasion of NSCLC cells via suppressing oncogenic B-cell lymphoma 6 protein expression levels (Sun *et al.*, 2016). Significant downregulation of miR-187-3p in hepatocellular carcinoma (HCC) tissues and cell lines was reported. Moreover, miR-187-3p expression was correlated with HCC metastasis and tumor, node and metastasis stages. Functional studies showed that miR-187-3p inhibited epithelial-mesenchymal transition of HCC cell lines and HCC metastasis *in vitro* and *in vivo* by targeting S100A4 (Dou *et al.*, 2016). These results indicate that miR-187-3p is novel tumor related microRNA. However, the role of miR-187-3p in infantile hemangioma remains unclear.

In the present study, we aim to study the participation and the underlying mechanisms of miR-187-3p in propranolol sensitivity of HemSCs.

## Materials and Methods

### Patients and clinical tissue samples

Total of 40 proliferating hemangiomas tissues and 20 normal skin tissues were obtained from Shanghai Ninth People’s Hospital. The blood samples were also collected from these patients. To use the tissues and blood for research, we obtained institutional ethics committee approval and patients’ informed consents. Written consent has been derived from all the participants. The experimental protocols were approved by the Ethics Committee of Shanghai Ninth People’s Hospital.

### HemSC isolation and culturing

HemSCs were isolated from proliferating-phase tissues (n=5, age (4.2±0.8 months)) with the addition digestion of Dispase II (D4693, Sigma, St. Louis, MO, USA). The isolated cells were then expanded in a rich endothelial growth medium (EGM-2; cc3162, with SingleQuot Kit Suppl. & growth factors, Lonza, Basel, Switzerland) supplemented with 20% fetal bovine serum (Invitrogen, Pleasanton, CA). Single expanded clone of HemSCs was prepared by a micromanipulation technique. Briefly, single cell was sucked by capillary pipette under the microscope, then cultured at 100 μl per well in 96-well dishes (Corning, Corning, NY). Cell proliferation was inspected and recorded for 3 whole weeks.

### RNA isolation, quantitative real-time PCR (qRT-PCR)

To isolate mRNAs from cells and tissues, TRIzol reagent (Invitrogen, Waltham, MA USA) was used according to manufacturer’s instruction. After blood collection, plasma was obtained by centrifugation (1200 g, 10 mins). Total RNA in plasma was isolated using Qiagen miRNeasy Serum/Plasma Kit according to the manufacturers’ instruction.

MiR-187-3p expression levels were detected by Taqman miRNA assay kit according to the protocol (Applied Biosystems, Waltham, MA, USA). RNU6B small nuclear RNA expression was used as control for miR-187-3p. GAPDH expression was used as control of Cyclin D1, Proliferating cell nuclear antigen (PCNA) and Nipped-B-like protein (NIPBL). The relative expression levels were detected by the 2^–ΔΔCT^ method.

Primers were used for qRT-PCR as follows: miR-187-3p: F 5'-CTT CGT GTC TTG TGT TGC-3', R 5'-GTG CAG GGT CCG AGG T-3'; U6: F 5'-TGC GGG TGC TCG CTT CGG CAG C-3', R 5'-GTG CAG GGT CCG AGG T-3'; Cyclin D1, F 5'-AAC TAC CTG GAC CGC TTC CT-3', R 5'-CCA CTT GAG CTT GTT CAC CA-3'; PCNA, F 5'-AGT GGA GAA CTT GGA AAT GGA A-3', R 5'-GAG ACA TGG AGT GGC TTT TGT-3'; NIPBL, F 5'-AGC AGA GAC CTG ATG GGC GA-3', R 5'-TGT CGC TCT GAT TCA CCC CTG-3'; GAPDH: F 5'-ACA ACT TTG GTA TCG TGG AAG G-3', R 5'-GCC ATC ACG CCA CAG TTT C-3'.

### Cell viability assay

Cell Counting Kit-8 (CCK-8) was used to detect cell viability according to the manufacturer’s protocol. Briefly, total of 1×10^4^ cells/well were seeded into 24-well plate. Seventy-two h later, 10 μl CCK-8 was added into every well, followed by 2 h 37°C incubation. Absorbance at 450 nm/630 nm was detected using a universal microplate reader.

### Western blot

Cells in culture were lysed by radio-immunoprecipitation (RIPA) assay along with the protease inhibitor cocktail. The concentrations of proteins were detected by BCA protein assay according to the manufacturer’s protocol. Samples were separated and electrophoresed by a 10% sodium dodecyl sulfate-poly-acrylamide-polyacrylamide gel electrophoresis gel. Then, the proteins were transferred to Transfer Membrane. The membranes were incubated with primary antibodies overnight at 4°C. Primary antibodies used in this study are as follows: anti-Cyclin D1 (1:1000), anti-PCNA (1:600), anti-NIPBL (1:500), anti-Cyclin D1 (1:1000), anti-ACTIN (1:2000). All the primary antibodies were from cell signaling technology. Then, the membranes were blocked with 5% no-fat milk for 1 h, followed by incubation with HRP conjugated secondary antibodies at room temperature for 1 hour. The signals were detected by chemiluminescence reaction. ACTIN was used as control.

### Caspase-3 Activity Assay

Caspase-3 activity was detected using Caspase-3 assay kit according to the manufacturer’s protocol. Briefly, cells were lysed by 50 μl lysis buffer for 10 mins. Then, the protein concentration in supernatant was detected by BCA protein assay. Total of 100 μg proteins in 50 μl lysis buffer were added into 96-well plate. Then, reaction buffer, DL-dithiothreitol and caspase-3 catalytic substrate DEVD-pNA was added, followed by incubation for 2 h at 37°C. Then, OD405 value was detected using microplate reader. The Caspase-3 activity was calculated according to the manufacturer’s protocol.

### Vector construction

The NIPBL was sub-cloned into pBabeMNires vector. Then, the construct was confirmed by sequencing. The primers used for CDS of NIPBL were as follows: F, 5'-CTA GAA TTC ATG AAT GGG GAT ATG CC-3', R, 5'-CTA GGA TCC TTA CGT AAT ACG CTG CGA AAT-3'. HemSCs cells were infected with NIPBL overexpression plasmids. Then, stable cells were established using antibiotic selection.

### Luciferase assay

HemSCs cells were added into 24-well plates at 37°C for 24 h. Then, the pmirGLO report vectors with WT 3'-UTR or MT 3'-UTR of NIPBL was co-transfected with miR-NC or miR-187-3P into HemSCs. HemSCs cells were lysed after culturing for 48 h, followed by detected by Dual-luciferase Reporter System. The primers of WT 3'-UTR or MT 3'-UTR of NIPBL used as follows. WT 3'-UTR: F, 5'-CTA CTC GAG AAT GAT TTT TAT GTG CT-3', R, 5' CTA GCG GCC GCT AAA CTA TAG TTT CTT T-3'. MT 3'-UTR: F, 5'-TAT CAA TAA GAG TAA CGC CTC TG-3', R, 5' CAG AGG CGT TAC TCT TAT TGA TA-3'.

### Biotin-miRNA pulldown assay

HemSCs cells were first washed using phosphate buffer saline, and treated with Tris-HCl buffer along with RNase Inhibitor and protease inhibitor cocktail. After incubation on ice for 20 mins, the cells were centrifuged (12,000 *g*, 15 mins, 4°C) for supernatant collection. Biotinylated RNA of miR-187-3p (biotin-miR-187-3p) or negative control (biotin-miR-NC) were added into 500 μl collected supernatant, followed by incubation for 30 mins at 4°C with 8 rpm shaking. Then, the supernatant with biotinylated RNAs were shaked at 30 rpm for 1 hour at 30°C. Total of 10 μL Streptavidin Mutein Matrix was added into the extract, and incubated at 4°C for 1 hour. Then, relative enrichment of NIPBL mRNA levels was detected in collected Streptavidin/biotin-miRNA/mRNA complex.

### Statistical analysis

All data was presented as the mean±SD from three independent experiments. The statistical significance was determined using one or two-way ANOVA or Student’s t-test. It was considered to be statistically significant when p value is less than 0.05.

## Results

### MiR-187-3p expression decreased and associated with propranolol resistance in infantile hemangiomas

**Fig. 1:**
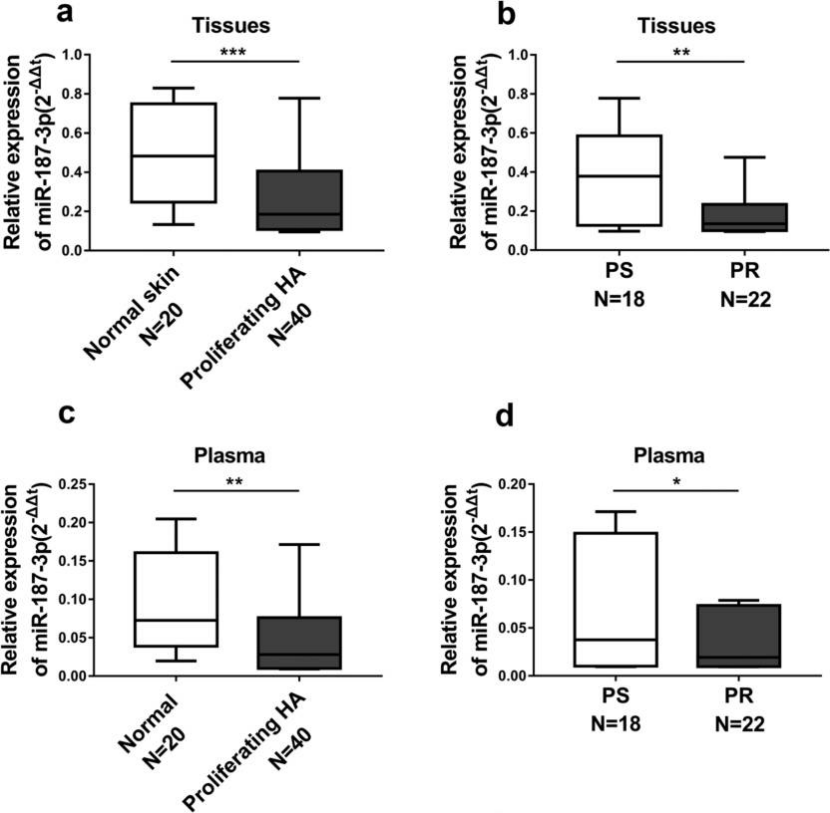
MiR-187-3p was downregulated and associated with propranolol resistance in hemangiomas. (a) qRT-PCR analysis of the expression levels of miR-187-3p in normal skin tissues (Normal skin) and proliferating hemangiomas tissues (Proliferating HA). (b) The miR-187-3p expression levels were much lower in hemangiomas tissues of propranolol-resistant patients (PR) than propranolol-sensitive patients (PS) quantified by qRT-PCR analysis. (c) qRT-PCR analysis of the expression levels of miR-187-3p in the plasma from the normal control (Normal) and proliferating hemangiomas patients (Proliferating HA). (d) The miR-187-3p expression levels were much lower in the plasma from propranolol-resistant patients (PR) than propranolol-sensitive patients (PS) quantified by qRT-PCR analysis. The data represent the mean±SD from three independent experiments. *P<0.05; **P<0.01; ***P<0.001. Student’s t-test.

To explore the biological role of miR-187-3p in infantile hemangiomas, we first determined the expression levels of miR-187-3p in 20 normal skin tissues (normal skin) and 40 proliferating hemangiomas tissues (proliferating HA). As shown in Fig. 1a, miR-187-3p expression level in normal skin tissues was much lower than proliferating HA tissues. In 40 proliferating HA samples, there are 18 propranolol-sensitive (PS) and 22 propranolol-resistant (PR) patients. We further determined miR-187-3p expression in the two groups. MiR-187-3p expression level in PR tissues was significantly inhibited compared with that in PS tissues (Fig. 1b).

MiR-187-3p expression level in plasma of proliferating HA patients was much lower than normal control (Fig. 1c). Moreover, in plasma of PR patients, miR-187-3p expression level was much lower than that in PS patients (Fig. 1d). These results indicate that miR-187-3p may serve as tumor suppressor in infantile hemangiomas and associated with propranolol resistance.

### MiR-187-3p inhibited propranolol resistance of HemSCs

**Fig. 2:**
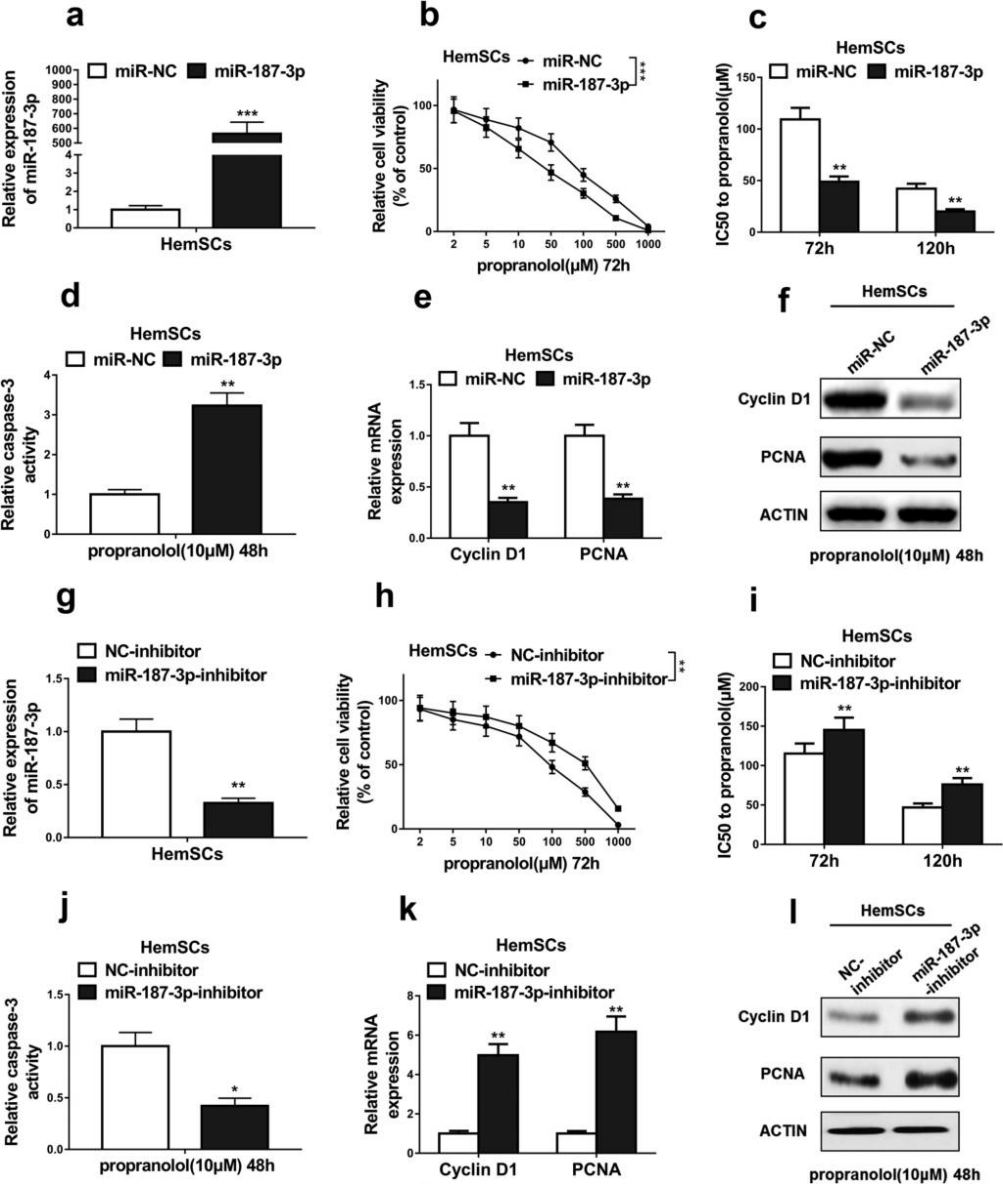
MiR-187-3p promoted propranolol sensitivity of HemSCs. (a) The overexpression efficiency of miR-187-3p in HemSCs transfected with miR-187-3p mimics (miR-187-3p) or mimics negative control (miR-NC) was confirmed by qRT-PCR analysis. (b) Cell viability was assessed by CCK-8 assay, HemSCs transfected with miR-187-3p mimics (miR-187-3p) or mimics negative control (miR-NC) were exposed to the indicated concentration of propranolol for 72 h. (c) IC_50_ values of propranolol following 72 h and 120 h treatment were measured after HemSCs were transfected with miR-187-3p mimics (miR-187-3p) or mimics negative control (miR-NC). (d) The caspase-3 activity of HemSCs transfected with miR-187-3p mimics (miR-187-3p) or mimics negative control (miR-NC) was measured after HemSCs were exposed to 10 μM propranolol for 48 h. (e and f) The expression levels of two important proliferative markers (Cyclin D1 and PCNA) of HemSCs were detected by qRT-PCR and western blotting, HemSCs transfected with miR-187-3p mimics (miR-187-3p) or mimics negative control (miR-NC) were exposed to 10 μM propranolol for 48 h. (g) The knockdown efficiency of miR-187-3p in HemSCs transfected with miR-187-3p inhibitor (miR-187-3p-inhibitor) or inhibitor negative control (NC-inhibitor) was confirmed by qRT-PCR analysis. (h) Cell viability was assessed by CCK-8 assay, HemSCs transfected with miR-187-3p inhibitor (miR-187-3p-inhibitor) or inhibitor negative control (NC-inhibitor) were exposed to the indicated concentration of propranolol for 72 h. (i) IC_50_ values of propranolol following 72 h and 120 h treatment were measured after HemSCs were transfected with miR-187-3p inhibitor (miR-187-3p-inhibitor) or inhibitor negative control (NC-inhibitor). (j) The caspase-3 activity of HemSCs transfected with miR-187-3p inhibitor (miR-187-3p-inhibitor) or inhibitor negative control (NC-inhibitor) was measured after HemSCs were exposed to 10 μM propranolol for 48 h. (k and l) The expression levels of two important proliferative markers (Cyclin D1 and PCNA) of HemSCs were detected by qRT-PCR and western blotting, HemSCs transfected with miR-187-3p inhibitor (miR-187-3p-inhibitor) or inhibitor negative control (NC-inhibitor) were exposed to 10 μM propranolol for 48 h. The data represent the mean±SD from three independent experiments. *P<0.05; **P<0.01; ***P<0.001 (two-way ANOVA for b and h, student’s t-test for others).

HemSCs were believed to be crucial for infantile hemangioma development and propranolol resistance. As miR-187-3p is corrected with propranolol resistance, we next detected the role of miR-187-3p in propranolol resistance of HemSCs. MiR-187-3p overexpression in HemSCs was first confirmed by qRT-PCR analysis (Fig. 2a). HemSCs transfected with miR-187-3p mimics (miR-187-3p) or mimics negative control (miR-NC) were exposed to different dose of propranolol for 72 h. As shown in Fig. 2b, cell viability assay was significantly inhibited after miR-187-3p overexpression. IC50 values of propranolol at 72 h and 120 h were much lower in HemSCs with miR-187-3p overexpression than miR-NC (Fig. 2c). Cell apoptosis of HemSCs induced by 10 μM propranolol for 48 h was enhanced by miR-187-3p overexpression, as evidenced by significantly higher caspase-3 activity in miR-187-3p-HemSCs than miR-NC-HemSCs (Fig. 2d). Moreover, miR-187-3p overexpression obviously inhibited HemSCs proliferation, as Cyclin D1 and PCNA expression significantly decreased in miR-187-3p-HemSCs at both mRNA and protein levels (Fig. 2e, 2f).

On the other hand, we further confirmed our results using miR-187-3p inhibitor. As shown in Fig. 2g, miR-187-3p inhibition in HemSCs was first verified by qRT-PCR analysis. Totally opposite to the effect of miR-187-3p overexpression on HemSCs, miR-187-3p inhibition promoted cell viability (Fig. 2h), increased IC50 values of propranolol (Fig. 2i), inhibited caspase-3 activity (Fig. 2j), enhanced Cyclin D1 and PCNA expression both on mRNA (Fig. 2k) and protein (Fig. 2l) levels. These results indicate that miR-187-3p promoted propranolol sensitivity of HemSCs.

### NIPBL was the direct target of miR-187-3p in HemSCs

**Fig. 3:**
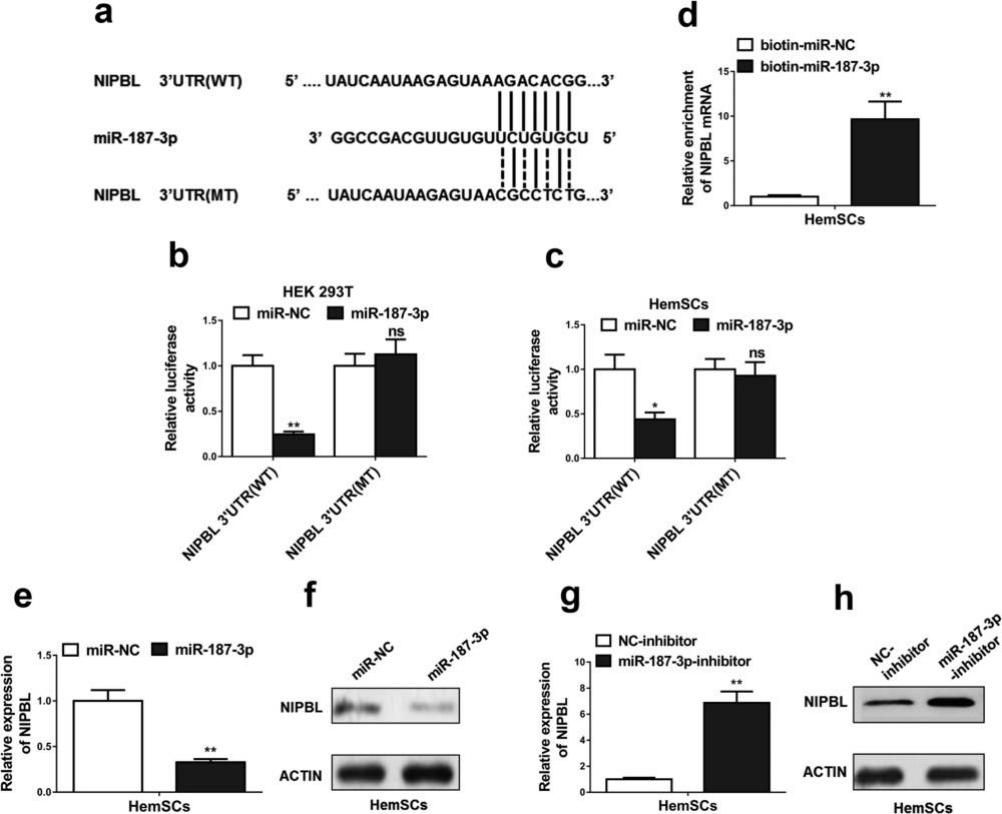
NIPBL was a direct target of miR-187-3p in HemSCs. (a) Schematic diagram of miR-187-3p binding sites in the NIPBL 3'UTR. Sequences were compared between the mature miR-187-3p and wild-type (NIPBL 3'UTR (WT)) or mutant (NIPBL 3'UTR (MT)) putative target sites in the 3'UTR of NIPBL. (b and c) Luciferase reporter assay was performed in HEK 293T and HemSCs co-transfected with plasmid containing NIPBL 3'UTR (WT) or NIPBL 3'UTR (MT) and miR-187-3p or miR-NC. (d) Detection of NIPBL mRNAs in biotinylated miRNA/target mRNA complex by real-time RT-PCR. The relative levels of NIPBL mRNA in the complex pulled down by using biotinylated miR-187-3p were compared to that of the complex pulled down by using the biotinylated control random RNA. (e–h) The relative expression levels of NIPBL in HemSCs transfected with indicated microRNA mimics and microRNA inhibitors or their respective negative controls detected by qRT-PCR and western blotting. The data represent the mean±SD from three independent experiments. *P<0.05; **P<0.01. Student’s t-test.

To further explore how miR-187-3p plays biological role in HemSCs, we predicted the target of miR-187-3p using bioinformatic algorithms, Targetscan (http://www.targetscan.org/vert_71/). NIPBL was predicted as the potential target of miR-187-3p and the predicted binding sites between miR-187-3p and wild-type (NIPBL 3'UTR (WT)) or mutant (NIPBL 3'UTR (MT)) were illustrated in Fig. 3a. The fragments containing miR-187-3p binding sites of NIPBL 3'UTR (WT) or NIPBL 3'UTR (MT) was cloned into luciferase reporter vector. MiR-187-3p overexpression significantly inhibited luciferase activity as compared with miR-NC, while the difference of luciferase activity between miR-187-3p and miR-NC disappeared in HEK 293 T cells (Fig. 3b) or HemSCs (Fig. 3c) with NIPBL 3'UTR (MT). Moreover, NIPBL mRNA expression level was significantly increased in complex pulled down by biotinylated miR-187-3p (biotin-miR-187-3P) as compared with that of iotinylated control random RNA (biotin-miR-NC) (Fig. 3d). In miR-187-3P transfected HemSCs, NIPBL expression significantly decreased at both mRNA and protein levels (Fig. 3e, 3f). Meanwhile, miR-187-3P inhibitor promoted NIPBL mRNA and protein levels (Fig. 3g, 3h). All the data suggeste that miR-187-3p directly targets NIPBL.

### Downregulation of NIPBL inhibited propranolol resistance of HemSCs

**Fig. 4:**
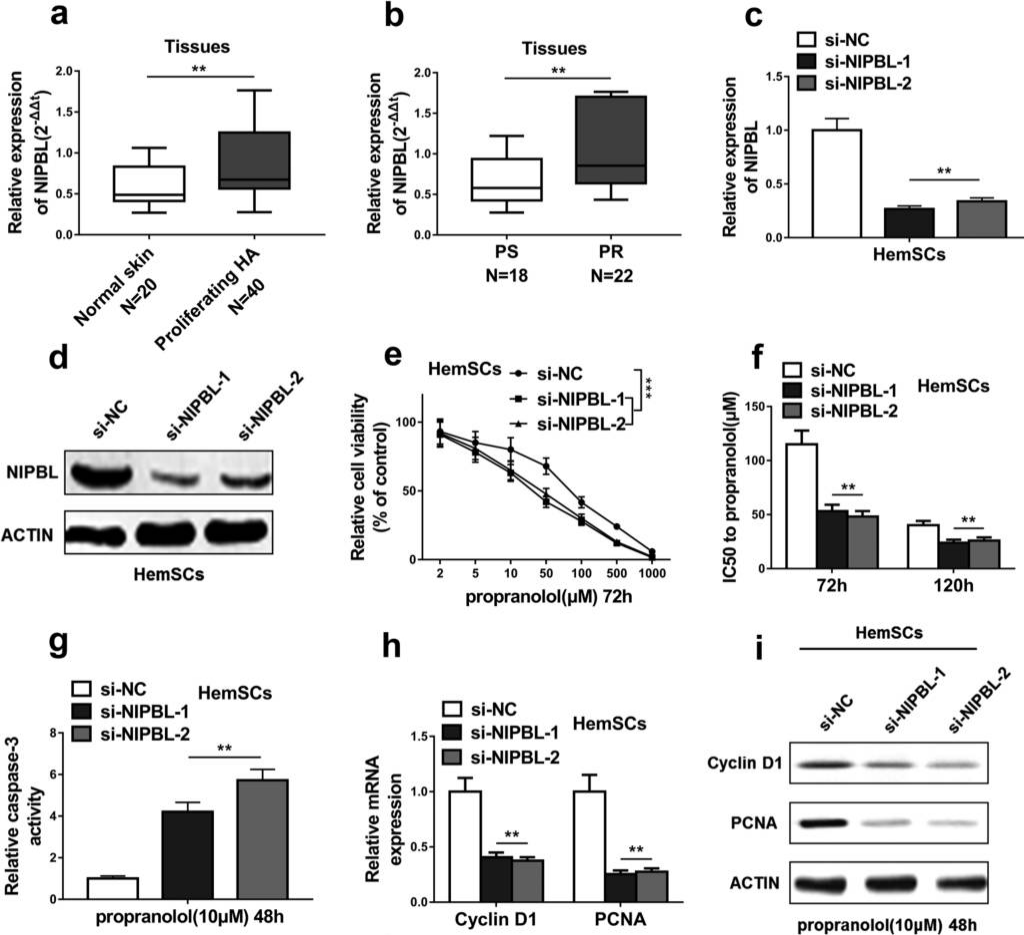
Downregulation of NIPBL inhibited propranolol resistance of HemSCs. (a) qRT-PCR analysis of the expression levels of NIPBL in normal skin tissues (Normal skin) and proliferating hemangiomas tissues (Proliferating HA). (b) The NIPBL expression levels were much higher in hemangiomas tissues of propranolol-resistant patients (PR) than propranolol-sensitive patients (PS) quantified by qRT-PCR analysis. (c and d) Knockdown efficiency of NIPBL in HemSCs transfected with NIPBL siRNA (si-NIPBL-1 and si-NIPBL-2) or negative control siRNA (si-NC) was confirmed by qRT-PCR and western blotting. (e) Cell viability was assessed by CCK-8 assay, HemSCs transfected with NIPBL siRNA (si-NIPBL-1 and si-NIPBL-2) or negative control siRNA (si-NC) were exposed to the indicated concentration of propranolol for 72 h. (f) IC_50_ values of propranolol following 72 h and 120 h treatment were measured after HemSCs were transfected with NIPBL siRNA (si-NIPBL-1 and si-NIPBL-2) or negative control siRNA (si-NC). (g) The caspase-3 activity of HemSCs transfected with NIPBL siRNA (si-NIPBL-1 and si-NIPBL-2) or negative control siRNA (si-NC) was measured after HemSCs were exposed to 10 μM propranolol for 48 h. (h and i) The expression levels of two important proliferative markers (Cyclin D1 and PCNA) of HemSCs were detected by qRT-PCR and western blotting, HemSCs transfected with NIPBL siRNA (si-NIPBL-1 and si-NIPBL-2) or negative control siRNA (si-NC) were exposed to 10 μM propranolol for 48 h. The data represent the mean±SD from three independent experiments. *P<0.05; **P<0.01; ***P<0.001 (two-way ANOVA for e, student’s t-test for others).

As NIPBL is the direct target of miR-187-3p, we next detected the influence of NIPBL on propranolol resistance of HemSCs. NIPBL expression levels were much higher in Proliferating HA than Normal skin tissues (Fig. 4a). Moreover, NIPBL expression significantly increased in RP tissues compared with PS tissues (Fig. 4b), indicating the potential role of NIPBL in propranolol resistance. To further verify our hypothesis, NIPBL was first downregulated by siRNA (si-NIPBL-1, si-NIPBL-2) and confirmed by qRT-PCR (Fig. 4c) and western blot (Fig. 4d). Downregulation of NIPBL significantly inhibited HemSCs cell viability after propranolol treatment (Fig. 4e). IC50 values of propranolol in HemSCs at two time points were much lower after NIPBL downregulation (Fig. 4f). Cell apoptosis of HemSCs, indicated by caspase-3 activity, significantly increased after transfected with si-NIPBL-1 or si-NIPBL-2 as compared with si-NC (Fig. 4g). In addition, NIPBL downregulation greatly inhibited cell proliferation of HemSCs, evidenced by decreased Cyclin D1 and PCNA expression at both mRNA and protein levels (Fig. 2h, 2i). These results indicate that NIPBL downregulation inhibited propranolol resistance of HemSCs.

### Re-introduction of NIPBL abolished the effects of miR-187-3p on HemSCs propranolol resistance

**Fig. 5:**
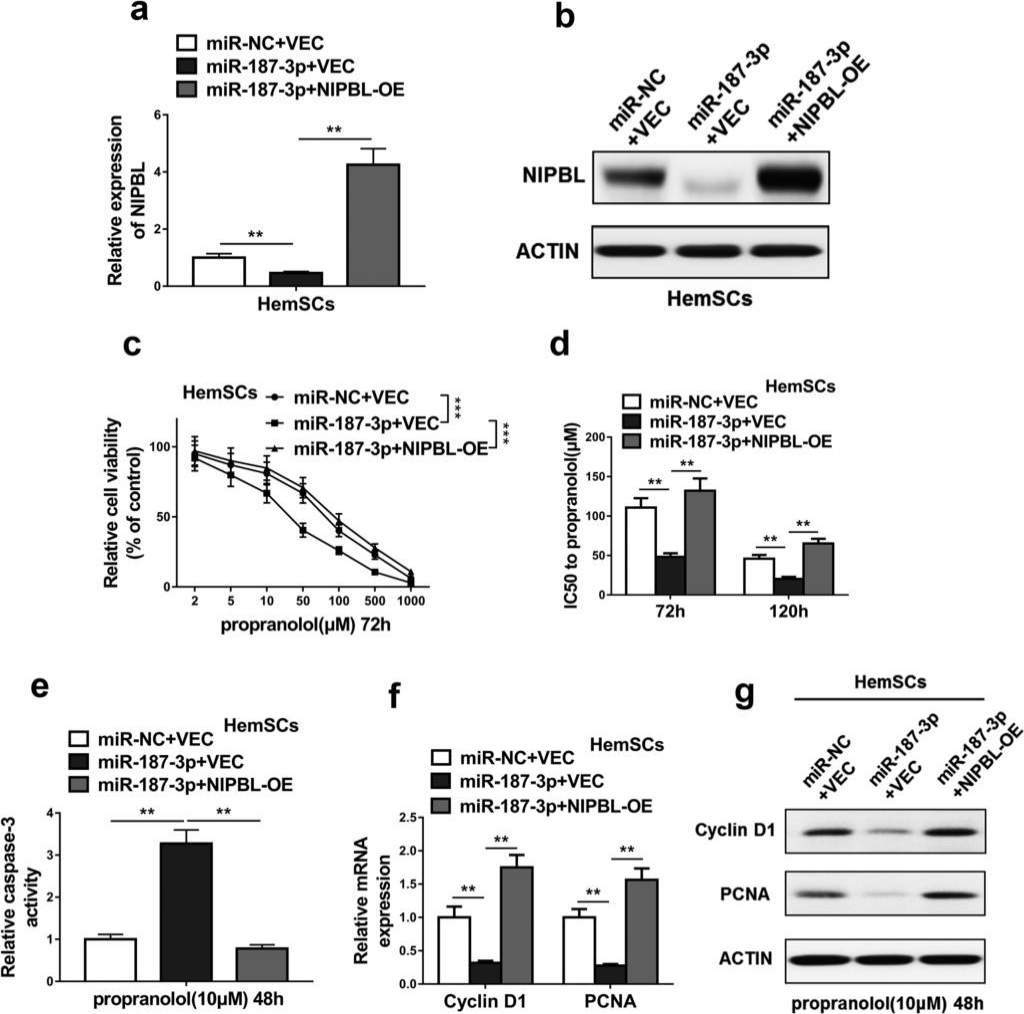
NIPBL mediated the effects of miR-187-3p on HemSCs propranolol resistance. (a and b) The expression levels of NIPBL were measured by qRT-PCR and western blotting in HemSCs co-transfected with mimics negative control and empty vector (miR-NC+VEC), miR-187-3p mimics and empty vector (miR-187-3p+VEC) or miR-187-3p mimics and NIPBL overexpression plasmid (miR-187-3p+ NIPBL-OE). (c) Cell viability was assessed by CCK-8 assay, HemSCs following the same co-transfection were exposed to the indicated concentration of propranolol for 72 h. (f) IC_50_ values of propranolol following 72 h and 120 h treatment were measured after HemSCs following the same co-transfection. (g) The caspase-3 activity of HemSCs following the same co-transfection was measured after HemSCs were exposed to 10 μM propranolol for 48 h. (h and i) The expression levels of two important proliferative markers (Cyclin D1 and PCNA) of HemSCs were detected by qRT-PCR and western blotting, HemSCs following the same co-transfection were exposed to 10 μM propranolol for 48 h. The data represent the mean±SD from three independent experiments. *P<0.05; **P<0.01; ***P<0.001 (two-way ANOVA for c, student’s t-test for others).

To further explore whether the effects of miR-187-3p on HemSCs propranolol resistance was attributable to decreased NIPBL expression, we restored the NIPBL expression in miR-187-3p-overexpressing HemSCs (miR-187-3p+NIPBL-OE), while miR-187-3p+VEC used as control. Assays of NIPBL expression by qRT-PCR and western blot showed successful re-introduction of NIPBL (Fig. 5a, 5b). Re-introduction of NIPBL decreased cell viability of miR-187-3p-overexpressing HemSCs (Fig. 5c). The IC50 values of propranolol in HemSCs at different time points increased after restoring NIPBL expression (Fig. 5d). Moreover, re-introduction of NIPBL inhibited cell apoptosis (Fig. 5e), promoted cell proliferation of HemSCs (Fig. 5f, 5g). These results indicate that NIPBL mediated the effects of miR-187-3p on HemSCs propranolol resistance.

## Discussion

Propranolol was considered to be safe and highly effective to infantile hemangioma therapy, and widely used in clinic (Drolet *et al.*, 2013). However, few infantile hemangioma patients showed resistance to propranolol treatment. In addition, hemangioma derived stem cells were believed crucial for infantile hemangioma development (Greenberger *et al.*, 2010a; Khan *et al.*, 2008), which promoted us to determine the mechanism and explore the possibility of how to decrease propranolol resistance, or enhance propranolol resistance sensitivity of hemangioma derived stem cells. In the present study, we found miR-187-3p enhances propranolol sensitivity of hemangioma stem cells via targeting NIPBL. To the best of our knowledge, this is the first study to report the role of miR-187-3p in infantile hemangioma, and how miR-187-3p plays the biological role to enhance propranolol sensitivity of hemangioma derived stem cells. This study linked microRNA, hemangioma derived stem cells and oncogene in infantile hemangioma, indicating that miR-187-3p may serve as a novel prognostic indicator and potential target for infantile hemangioma therapy.

HemSCs were identified and selected using CD133 in a mouse *in vivo* model (Khan *et al.*, 2008). The selected HemSCs expressed VEGF-R1, VEGF-R2, and CD90 (a mesenchymal cell marker). In immunodeficient mice, HemSCs form human CD31^+^GLUT-1^+^ (glucose transporter 1) blood vessels during 7–14 days, which could form blood vessels again in the secondary recipients. In addition, human adipocytes were also formed after 28 days. *In vitro* study indicated the ability of highly proliferation and differentiation into various cell types. All of these findings indicate that HemSCs are the precursors of infantile hemangioma. Dexamethasone, one kind of corticosteroids, was another common choice for infantile hemangioma in clinic. Previous study reported that dexamethasone inhibited vasculogenesis of HemSCs in mouse model in a dose-dependent manner. Mechanically, dexamethasone suppressed VEGF-A expression levels of HemSCs, while downregulation of VEGF-A by short hairpin RNA reduced vasculogenesis of HemSCs *in vivo*. Moreover, expression levels of other proangiogenic factors, such as matrix metalloproteinase 1 and interleukin-6 were also were found to be suppressed in HemSCs after corticosteroid treatment (Greenberger *et al.*, 2010b), indicating that HemSCs were the potential target for infantile hemangioma treatment. In our present study, we treated HemSCs using propranolol. HemSCs apoptosis induced by propranolol increased after miR-187-3p overexpression. Moreover, miR-187-3p overexpression inhibited cell viability and cell proliferation of HemSCs, indicating miR-187-3p promoted propranolol sensitivity of HemSCs.

NIPBL is the human homolog of evolutionarily conserved cohesin loading factor SCC2, and found in nuclear of all eukaryotic cells (Krantz *et al.*, 2004; Tonkin *et al.*, 2004). NIPBL plays crucial role in various biological processes via interacting with cohesin complex (Gause *et al.*, 2008). In 120 Cornelia de Lange syndrome patients, about 47% (56/120) of all cases showed NIPBL mutations (Gillis *et al.*, 2004). NIPBL was also reported to be important for tumor development. In breast cancer cell lines, NIPBL expression increased at both mRNA and protein levels. Downregulation of NIPBL arrested cell cycle into G0/G1 phase and promoted cell apoptosis (Zhou *et al.*, 2017). NIPBL mutation was also found in 91 gastric cancer and 100 colorectal cancer patients when using PCR-based single strand conformation polymorphism assay, indicating the correlation of NIPBL between gastric and colorectal cancer (Kim *et al.*, 2013). NIPBL expression in detected by immunochemistry in 123 lung adenocarcinoma samples displayed a strongly positive correlation with poor prognosis of non-small cell lung cancer patients. Moreover, high expression of NIPBL was associated lymph node metastasis and tumor differentiation. Cell proliferation, migration and invasion were significantly inhibited after downregulation of NIPBL. More importantly, NIPBL downregulation improved chemotherapy resistance (cisplatin, paclitaxel, gemcitabine hydrochloride) of non-small cell lung cancer cells (Xu *et al.*, 2015). Mechanically, NIPBL downregulation promotes autophagy and impairs DNA damage response of non-small cell lung cancer cells (Zheng *et al.*, 2018), indicating that NIPBL may serve as a therapeutic target of chemotherapy resistance. Consistent with previous study, we found NIPBL expression levels increased in PR patients compared with PS patients. NIPBL downregulation inhibited cell viability, promoted cell apoptosis, while re-introduction of NIPBL showed the opposite effects on cell viability and cell apoptosis. These results indicate that NIPBL may be a potential target for propranolol resistance of hemangioma derived stem cells.

Taken together, we found that miR-187-3p expression levels were down-regulated in infantile hemangioma tissues and promoted propranolol sensitivity of HemSCs via targeting NIPBL, which showed increased expression in infantile hemangioma, especially propranolol resistance patients. MiR-187-3p may serve as a novel prognostic indicator and potential target for infantile hemangioma therapy.

## Conclusion

In the current study, we confirm that miR-187-3p participates in propranolol sensitivity of hemangioma stem cells. Its expression is down-regulated in infantile hemangioma tissues, and *in vitro* studies suggest that it promotes propranolol sensitivity of HemSCs. Mechanistic investigation reveals that the possible target of miR-187-3p is NIPBL. Our study suggests that miR-187-3p may serve as a novel prognostic indicator and potential target for infantile hemangioma therapy.

## Acknowledgments

This study is financially supported by the grants of National Natural Science Foundation of China (Nos. 81771087), and Shandong Provincial Natural Science Foundation, China (No. ZR2017BH005).

## Conflict of interest

The authors declare that there is no conflict of interests.
